# Dr. Pavy's Researches on Diabetes

**Published:** 1894-07-28

**Authors:** 


### Dr. Pavy's Researches on Diabetes.

In the admirable Croonian Lectures recently delivered before the Royal College of Physicians, Dr. Pavy has formulated his objections to the doctrine of glycogenesis, which, springing from the observations of Bernard, has been largely accepted by physiologists. But he has done more. His researches have not aimed merely at the destruction of what he considers an erroneous dogma, but have brought to light a string of evidence which has made it possible to construct a theory explanatory both of the intimate processes of physiological digestion and of the pathological processes of diabetes; hence his title, "A New Departure in Diabetes." For the better understanding of the matter let us recapitulate. The usually accepted theory is that carbohydrate food is absorbed as sugar, which is partly used at once, by muscle for example, and partly stored in the form of glycogen to meet future requirements.* "Sugar is a constant constituent of the blood, which in a starving or a well-nourished animal alike contains about 1 per 1,000 of sugar. The amount of sugar present in the venous blood of muscle is constantly slightly below that present in arterial blood, and the amount present is at its minimum in the venous blood of active muscle. Sugar is therefore consumed by living muscle, especially during activity. The amount of sugar present in hepatic venous blood is constantly slightly above that present in portal venous blood. Sugar is therefore produced by the liver. We have now no difficulty in following the sugar cycle in an active well fed animal. Absorbed sugar enters the blood, is to a great extent stored as glycogen in the liver, is consumed by living muscle, is discharged as $CO_2$ and as $H_2O$. The part played by glycogen is that of temporary carbohydrate reserve."

As an almost necessary corollary to this is the doctrine that the kidney has a certain tolerance of sugar, so that unless the quantity in the blood rises beyond a certain amount it is not passed out by the urine.

To all this, except the turning of sugar into glycogen, Dr. Pavy gives a direct denial, and it will be noted that his conclusions are not the result merely of fresh interpretations of old facts, but are founded on an absolute traversing of the facts themselves. This is important. Disputations as to theory are apt to be endless, but when the experiments themselves are brought in question, it becomes merely a matter of fact as to which is right, and it ought not to be difficult by repeating the experiments to arrive at certainty.

Bernard's views as to glycogenesis hinge largely on the "fact" that there is more sugar in the blood coming from the liver than in the portal vein. This Dr. Pavy absolutely denies, stating as the result of numerous observations that the blood flowing from the liver is not more saccharine than that flowing to it, but the contrary. Again, we are taught by physiologists that the sugar is consumed in living muscle, but Dr. Pavy says that as a matter of experimental observation it does not thus disappear, although he does admit that in his experiments, so far as any difference was discovered between venous and arterial blood, the excess of sugar was on the arterial side, but only to the minute extent of 0·003 per 1,000. Again, so far from the kidney tolerating sugar up to a certain point, Dr. Pavy's experiments failed to support this proposition of Bernard's; on the contrary, even small amounts of sugar in the general circulation were not tolerated, but were passed off by the kidneys.

* Waller's "Human Physiology."

These organs are, according to Dr. Pavy, a most delicate and sensitive indicator of the amount of sugar in the blood, urine always containing sugar, small in amount, but corresponding with the small quantity in the blood.

Dr. Pavy's view of the digestion of proteids and carbohydrates may be summarised as follows: By the action of chemical agents and ferments in the digestive canal both proteid food and insoluble carbohydrates are changed. The complex starch molecule is hydrated and broken down into the smaller molecules of, at first, dextrine and maltose, and, finally, maltose alone. The proteid molecule is also broken down into albumoses, peptone, and sugar, and then is built up again by the protoplasm of the intestinal villi into the proteids of the body.

This is the central fact of Dr. Pavy's investigations and discoveries, viz., that proteids are glucoside bodies, and that while, under the action of ferments, sugar may be cleaved off as a by-product of their analysis, if one may so say, under the influence of protoplasmic or vital action a process of synthesis may take place, sugar being absorbed and incorporated with nitrogenous material to build up the proteid proper to the individual of whom the protoplasm forms a part. Dr. Pavy also shows that the villi possess the power of converting a portion of the carbohydrate food into fat.

Instead, then, of the simple but crude theory, according to which sugar is absorbed and used as such, we have to recognise that the carbon and the hydrogen of the carbohydrates are carried to the tissues for final consumption, not as loose sugar, but as part and parcel of the proteids in the lymph stream, and that energy is developed in muscles by the oxidation, not of sugar, but of the carbon contained in the proteids themselves. Roughly we may consider proteid matter as a carrier of carbon much in the same way as we know the hæmoglobin to be a carrier of oxygen. The protoplasm of the villi then is not only a builder of proteid but acts as a barrier to the entry of sugar as such. Still, by the blood vessels a certain quantity of sugar is absorbed and passes by the portal vein to the liver; here, however, it is stored as glycogen, to be ultimately converted, probably, into fat. The liver then instead of being a maker of sugar is another line of defence against the entry of sugar into the body, and as we know from Dr. Pavy's researches that in proportion as sugar exists in the blood it will flow out in the urine, the pathology of diabetes is driven back to a question of protoplasmic activity in the villi of the intestines on the one hand, and in the liver on the other. A diabetic is a person who has not protoplasmic power sufficient to dispose of the carbohydrates in his diet by synthesis to proteid, by transmutation into fat, and by dehydration into glycogen; and a confirmed diabetic in whom the strictest diet will not entirely stay the excretion of sugar, is one in whom this protoplasmic defect is so pronounced that even the sugar produced by the ferment-decomposition of proteid food cannot be entirely recombined in the production of living proteids.

It is too early to say how far Dr. Pavy's researches will directly influence treatment, but they certainly enable us to form a crisp and clear mental picture of the processes of normal assimilation, of the defects in those processes by which diabetes is produced, and especially of the mode in which glycosuria occurs in those cases in which it persists notwithstanding careful adherence to a purely proteid diet.

